# Œdème papillaire revelant une malformation d’Arnold Chiara type 1 : à propos d’un cas

**DOI:** 10.11604/pamj.2016.24.293.7415

**Published:** 2016-08-03

**Authors:** Mouhoub Imane, Maadane Asmae, Ramdani Toufik, Sekhsoukh Rachid

**Affiliations:** 1 Service d’Ophtalmologie, CHU Mohammed VI, Oujda, Maroc

**Keywords:** Arnold Chiari, œdème papillaire, microcorie, Arnold Chiari, papillary oedema, microcoria

## Abstract

La malformation d'Arnold Chiari de type 1 est définie par une hernie des tonsilles cérébelleuses dans le foramen magnum de plus de 5 mm. Les symptômes sont dominés par les céphalées surtout occipitales, les torticolis, et parfois des troubles de déglutition. Sur le plan ophtalmologique les anomalies de convergences, les paralysies oculomotrices et la diplopie sont les principaux signes cliniques retrouvés. Nous rapportons le cas d'un enfant de 9 ans, qui consulte pour une baisse d'acuité visuelle évoluant depuis 6 mois. L'examen ophtalmologique objective une acuité visuelle chiffrée à 4/10^ème^ aux deux yeux. Une motilité oculaire conservée ainsi qu'un nystagmus rotatoire. L'examen du segment antérieur montre une mégalocornée, sans goniodysgénésie, un iridodonesis associé à une atrophie du muscle dilatateur, et une microcorie avec un reflex photo-moteur paresseux. Le tonus oculaire est correct à 14 mmHg. Le fond d'œil, malgré la difficulté de le réaliser, objective la présence d'un œdème papillaire bilatéral stade II. L'examen général retrouve un torticolis, une scoliose et un syndrome tétra-pyramidal. L'imagerie par résonance magnétique a mis en évidence une malformation de CHIARI type I, associée à une hydrocéphalie et une syringomyélie. Une intervention neurochirurgicale reposant sur une dérivation interne du LCR avec décompression ostéodurale cervico occipitale est proposée. L'évolution est favorable avec une régression des signes cliniques. Sur le plan ophtalmologique, on note une régression de l'œdème papillaire, mais l'acuité visuelle est restée stationnaire. La survenue d'un œdème papillaire est rare dans la malformation de Chiari type 1, il n'a été décrit que chez 2% des patients symptomatiques. Sa physiopathologie est encore mal élucidée. L'originalité de notre observation consiste en l'association de malformations cérébelleuses avec des malformations oculaires à type de mégalocornée et de microcorie rendant l'examen ophtalmologique encore plus difficile à réaliser.

## Abstract (en)

Arnold Chiari malformation type 1 is defined as a herniation of the cerebellar tonsils into the foramen magnum of more than 5 mm. Symptoms are most commonly dominated by occipital headache, torticollis and sometimes swallowing disorders. Ophthalmologically abnormal convergences, oculomotor palsy and diplopia are the main clinical signs. We report the case of a 9 year old child, who presented with visual loss evolving since 6 months. Ophthalmologic examination showed visual acuity of 4/10 in both eyes, retained ocular motility and rotational nystagmus. The examination of the anterior segment of the eye showed megalocornea with no evidence of goniodysgenesis, iridodonesis associated with atrophy of the dilator muscle and microcoria with lazy photomotor reflex. Normal intraocular pressure was 14 mmHg. Ocular fundus examination, despite difficulties in performing it, objectified bilateral papilledema (stage II). General physical examination showed torticollis, scoliosis and a tetra-pyramidal syndrome. MRI showed Chiari malformation type I associated with hydrocephalus and syringomyelia. Neurosurgical intervention based on internal CSF drainage with occipitocervical osteo-dural decompression was proposed. The evolution was favorable with regression of clinical signs. Ophthalmologically, there was a regression of papilledema but visual acuity remained stationary. The occurrence of papilledema associated with Chiari malformation type 1 is rare, it has been only reported in 2% of symptomatic patients. Its pathophysiology is still poorly understood. The originality of our study consists in the association of cerebellar malformations with ocular malformations including megalocornea and microcoria which make ophthalmologic examination more difficult to perform.

## Introduction

La malformation d'Arnold Chiari de type 1 est définie par une hernie des tonsilles cérébelleuses dans le foramen magnum de plus de 5 mm. Cette malformation entraine un déséquilibre de pression entre la cavité encéphalique et l'espace méningé spinal qui peut conduire une hydrocéphalie et une syringomyélie [1]. Les symptômes sont dominés par les céphalées surtout occipitales, les torticolis, et parfois des troubles de déglutition. Sur le plan ophtalmologique les anomalies de convergences, les paralysies oculomotrices et la diplopie sont les principaux signes cliniques retrouvés [2]. La présence d'un œdème papillaire est peu fréquente dans le syndrome de Chiari type 1. La particularité de notre observation c'est qu'en plus des malformations cérébelleuses, la patiente présente des anomalies malformatives oculaires rendant l'examen ophtalmologique encore plus difficiles à réaliser.

## Patient et observation

**Figure 1:**
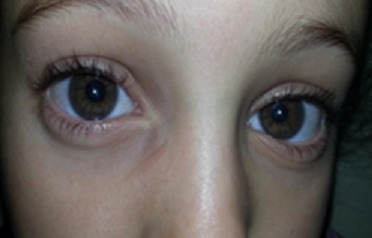
Mégalocornée avec microcorie

**Figure 2:**
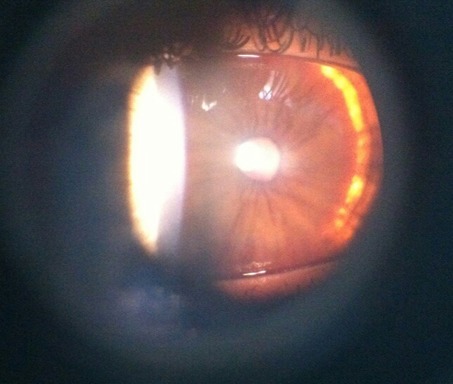
Atrophie du dilatateur de l’iris

**Figure 3:**
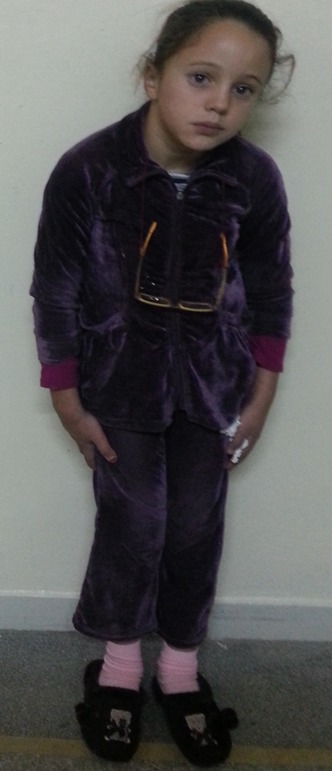
Scoliose avec torticolis

**Figure 4:**
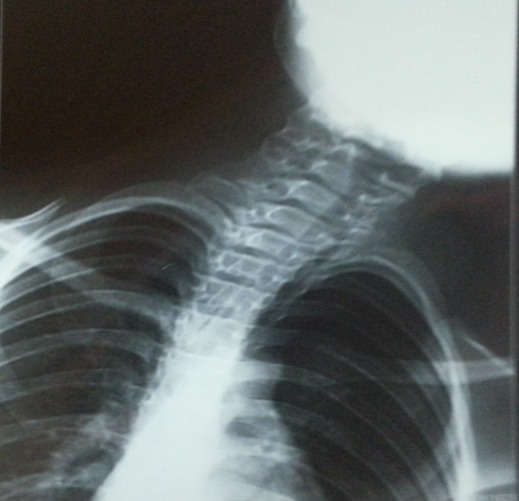
Scoliose a la radiographie thoracique

**Figure 5:**
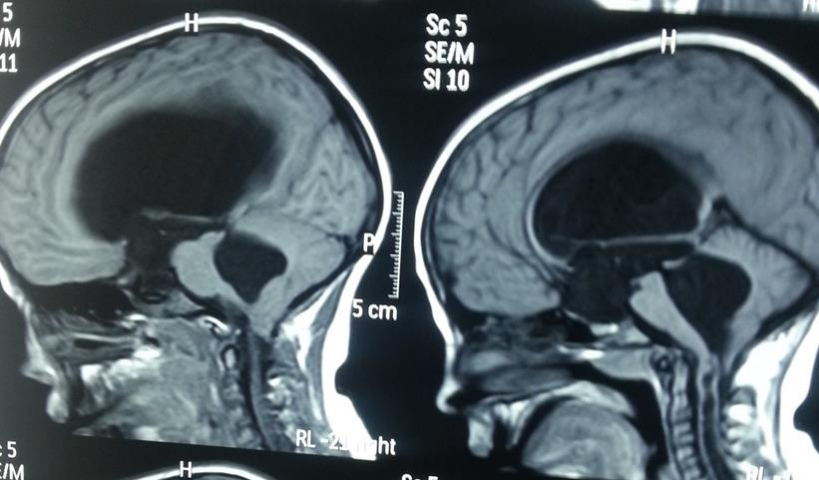
IRM cérébromédullaire: malformation de Chiari type I, associé à une hydrocéphalie et une syringomyélie

Nous rapportons le cas d'un enfant de 9 ans, qui consulte pour une baisse d'acuité visuelle évoluant depuis 6 mois. Il s'agit d'un enfant de 9 ans, de sexe féminin. Consulte pour un brouillard visuel, avec baisse de l'acuité depuis quelques mois. L'interrogatoire ne trouve pas de notion de consanguinité. Il révèle l'existence de céphalées depuis plusieurs mois sans notion d'amaurose ou de diplopie. L'examen ophtalmologique objective une acuité visuelle chiffrée à 4/10^ème^ aux deux yeux non améliorable par un essaie de correction, une motilité oculaire conservée ainsi qu'un nystagmus rotatoire. L'examen du segment antérieur montre une mégalocornée avec diamètre cornéen à 14 mm sans goniodysgénésie (Figure 1), un iridodonesis associé à une atrophie du muscle dilatateur, et une microcorie (Figure 2) avec un reflex photo-moteur paresseux. Le tonus oculaire est correct à 14 mmHg. Le fond d'œil met en évidence un œdème papillaire bilatéral stade II, sans autres anomalies rétiniennes ou vasculaires. L'examen général retrouve une scoliose thoracique à convexité droite et un torticolis (Figure 3). L'examen neurologique trouve des Paresthésies aux quatre membres avec une ataxie cérébelleuse. Il met également en évidence un syndrome tétra-pyramidal fait de troubles de la marche, une claudication motrice intermittente, des réflexes ostéotendineux vifs avec un babinski positif. La radiographie thoracique confirme la scoliose (Figure 4). Un scanner cérébral est réalisé rapidement en première intention. Sur les coupes horizontales passant dans le plan du trou occipital, une malformation de Chiari type I est fortement suspectée. Un examen IRM complète le bilan d'imagerie. En T1 sagittal, la malformation de Chiari type I est confirmée. Les amygdales cérébelleuses sont anormalement basses dans la partie haute du canal cervical et prennent un aspect effilé, Cette protrusion des amygdales cérébelleuses est mesurée à 10 mm sous la ligne basi occipital (Figure 5). Les coupes Axial FLAIR montrent une hydrocéphalie avec dilatation des cavités ventriculaires sus jacentes. Enfin l'exploration de l'IRM médullaire a permis de trouver une cavité syringomyélique associée. Devant la symptomatologie clinique et en présence de bilan morphologique rapportant une malformation Chiari type I avec complications sus et sous-jacente, la patiente a bénéficiée d'une prise en charge neurochirurgicale consistant à une dérivation interne du LCR avec décompression ostéo-durale cervico-occipitale. L'évolution est favorable avec une régression des signes cliniques, réapparition de la citerne cérébello-médullaire et diminution de la syringomyélie. Sur le plan ophtalmologique, on note une régression de l'œdème papillaire, mais l'acuité visuelle est restée stationnaire.

## Discussion

La malformation d'Arnold-Chiari (MAC) est une malformation congénitale du cervelet. Elle a été décrite pour la première fois en 1893 par un anatomopathologiste autrichien: Hans Chiari. Lors de ces observations post-mortem, Chiari a observé que les tonsilles cérébelleuses étaient anormalement basses et venaient s'engager au travers du foramen magnum. Quelques années plus tard, l'allemand Julius Arnold, contribua à donner la définition de cette maladie [3]. 4 types de malformation de sévérité croissante sont décrits. Le type 1 est défini par une hernie des tonsilles cérébelleuses dans le foramen magnum de plus de 5 mm. On parle d'ectopie lorsque la ptose est de 3 à 5 mm, moins de 3 mm est considérée comme une variante de la normale. Le type2 est caractérisé par une Ptose du vermis inférieur, de la protubérance et de la moelle allongée dans le canal cervical, à travers un foramen magnum élargi. Il est toujours associé à une myéloméningocèle. Dans le type 3 les malformations sont plus complexes, on note une hernie du cervelet dans un spina bifida occipito vertébral. Le type 4 est distingué par une hypoplasie du cervelet, associé à une ectopie bulbaire [3, 4]. En cas de malformation de Chiari de type 1, l'obstruction chronique du foramen magnum empêche la circulation physiologique du LCR dans le canal vertébral et crée une hyperpression dans les espaces sous arachnoïdiens, à l'origine d'une pénétration du LCR dans le canal épendymaire. Si le canal épendymaire présente une sténose incomplète, il se dilate globalement. Si le canal épendymaire est totalement sténosé, le LCR s'accumule alors dans le parenchyme médullaire, entraînant un état pré-syringomyélique puis une cavité syringomyélique dans environ 50% des cas [5]. La malformation de Chiari type 1 est plus fréquente chez la femme et sa prévalence peut être approchée à 1 sur 1280 individus. L'âge moyen d'apparition des symptômes est de 25 ans. Rare sont les cas découvert avant l'âge de 10 ans comme le cas de notre patiente. Elle est souvent asymptomatique, mais peut apparaitre sous forme de signes mineurs ou peu handicapant. Il s'agit de céphalées présentes dans un cas sur deux, qui peuvent revêtir des présentations variées. Ils sont particulièrement évocateurs lorsqu'ils sont postérieurs, déclenchés par la toux, l'éternuement, la manœuvre de Vasalva. Sont aussi typiques des vertiges déclenchés par les changements rapides de position de la tête. Une dysphonie ou une dysphagie peuvent apparaitre dans 5-15% des cas, associé à des acouphènes, une ataxie, des dysesthésies du tronc ou des extrémités. D'autres manifestations vont revêtir un caractère nettement plus grave comme une apnée du sommeil, des syncopes, des arrêts respiratoires, des morts subites pour des traumatismes mineurs, des tétraparésies transitoires [1, 2, 4]. On peut également retrouver des symptômes visuels ou oculomoteurs et notamment un nystagmus, une paralysie oculomotrice surtout de la 6^ème^ paire crânienne, ou une diplopie. Dans le cas de notre patiente, la baisse de l'acuité visuelle, constitue le principal motif de consultation, bien que la patiente présente d'autre signe de la maladie à savoir les céphalées, le nystagmus, le torticolis et la scoliose, mais elle n'avait jamais pu bénéficié d'un bilan radiologique complet probablement en raison de son niveau socio économique bas. La survenue d'un œdème papillaire est rare dans la malformation de Chiari type 1, il n'a été décrit que chez 2% des patients symptomatiques [6]. Sa physiopathologie est encore mal élucidée. Selon plusieurs auteurs [7, 8], ces patients présentent à cause de la malformation cérébelleuse une augmentation de la résistance à l'écoulement du LCR au niveau de la fosse postérieure modifiant ainsi l'effet amortissant de cette cavité, qui est à l'état normal, capable d'atténuer le volume veineux et les changements de pression qui se produisent au cours de la respiration physiologique des battements cardiaques, des changements de posture et la manœuvre de Valsalva. En effet, l'obstruction de l'écoulement du LCR au niveau du foramen magnum pourrait altérée l'équilibre de pression entre la cavité encéphalique et l'espace méningé spinal, conduisant ainsi à une augmentation de la pression intracrânienne et un œdème papillaire. Cependant, la survenue d'un œdème papillaire chez une proportion réduite des patients atteints de cette malformation, suggère l'existence d'autres anomalies structurales congénitales indépendamment des malformations cérébelleuses. La prise en charge de l'œdème papillaire secondaire à la malformation de Chiari 1 n'est pas bien codifiée. Vaphiades et al [2] ont décrit en 2002 une série de quatre malades présentant une malformation de Chiari de type 1 avec un œdème papillaire, chez qui la prescription de l'acétazolamide était inefficace, et pour qui la décompression de la fosse postérieure a permit une réduction des symptômes. Zhang et al [8] a présenté en 2011 le cas d'une patiente avec une malformation Chiari type 1 et un œdème papillaire, et qui a bénéficié d'une décompression sous occipitale, il a noté une diminution des signes cliniques mais la malade a gardé une atrophie optique bilatérale. En 2015, A. Rigamonti [9] a décrit le cas d'une femme de 40 ans qui présente un œdème papillaire sur malformation de Chiari 1 chez qui la décompression chirurgicale a permit une disparition complètes de signes cliniques et une régression l'œdème papillaire avec une bonne récupération visuelle. En réalité, à la découverte d'une malformation de Chiari type 1, le traitement chirurgical n'est pas toujours indiqué. Cependant la présence d'un œdème papillaire dont l'évolution pour se compliquer d'une perte visuelle, incite à prendre une décision multidisciplinaire de l'intervention chirurgicale [2, 8, 9]. Chez notre malade, la décompression ostéo-durale cervico-occipitale a permit une réduction des symptômes cliniques, et une régression de l'œdème papillaire mais sans amélioration de l'acuité visuelle. L'originalité de notre observation consiste en l'association de malformations cérébelleuses avec des malformations oculaires à type de mégalocornée et de microcorie rendant l'examen ophtalmologique encore plus délicat faire.

## Conclusion

La malformation d'Arnold Chiari type 1 est une pathologie peu fréquente. Elle peut se manifester par plusieurs signes cliniques. La découverte d'un œdème papillaire associé, suggère une intervention neurochirurgicale dans les plus brefs délais afin d'éviter l'évolution vers la perte de la fonction visuelle.
